# LRRC25 expression during physiological aging and in mouse models of Alzheimer’s disease and iPSC-derived neurons

**DOI:** 10.3389/fnmol.2024.1365752

**Published:** 2024-02-26

**Authors:** Dinesh Devadoss, Juliet Akkaoui, Madhavan Nair, Madepalli K. Lakshmana

**Affiliations:** Department of Cellular and Molecular Medicine, Herbert Wertheim College of Medicine, Florida International University, Miami, FL, United States

**Keywords:** aging, LRRC25, brain regions, Alzheimer’s disease, 3xTg mice, APΔE9 mice, iPSC neurons, immunohistochemistry

## Abstract

The leucine-rich repeat-containing protein 25 (LRRC25) is relatively a novel protein with no information on its role in neuronal or brain function. A recent study suggested LRRC25 is a potential risk factor for Alzheimer’s disease (AD). As a first step to understanding LRRC25’s role in the brain and AD, we found LRRC25 is expressed in both cell membranes and cytoplasm in a punctuate appearance in astrocytes, microglia, and neurons in cell lines as well as mouse brain. We also found that LRRC25 expression is both age- and brain region-dependent and that 1-day-old (1D) pups expressed the least amount of LRRC25 protein compared to adult ages. In the APΔE9 mice, immunoblot quantified LRRC25 protein levels were increased by 166% (**p < 0.01) in the cortex (CX) and by 215% (***p < 0.001) in the hippocampus (HP) relative to wild-type (WT) controls. Both the brainstem (BS) and cerebellum (CB) showed no significant alterations. In the 3xTg mice, only CX showed an increase of LRRC25 protein by 91% (*p < 0.05) when compared to WT controls although the increased trend was noted in the other brain regions. In the AD patient brains also LRRC25 protein levels were increased by 153% (***p < 0.001) when compared to normal control (NC) subjects. Finally, LRRC25 expression in the iPSC-derived neurons quantified by immunofluorescence was increased by 181% (**p < 0.01) in AD-derived neurons when compared to NC-derived neurons. Thus increased LRRC25 protein in multiple models of AD suggests that LRRC25 may play a pathogenic role in either Aβ or tau pathology in AD. The mechanism for the increased levels of LRRC25 in AD is unknown at present, but a previous study showed that LRRC25 levels also increase during neonatal hypoxic-ischemia neuronal damage. Based on the evidence that autophagy is highly dysregulated in AD, the increased LRRC25 levels may be due to decreased autophagic degradation of LRRC25. Increased LRRC25 in turn may regulate the stability or activity of key enzymes involved in either Aβ or hyperphosphorylated tau generation and thus may contribute to increased plaques and neurofibrillary tangles.

## Introduction

The risk of Alzheimer’s disease (AD) increases with age and thus age is the greatest risk factor for AD. Gender is another risk factor, as more women than men are diagnosed with AD. AD is the leading cause of cognitive impairment and dementia in individuals aged 65 years and older and may even affect as many as 30% of those aged 85 years or older (Atri, 2019; GBD 2019 Dementia Forecasting Collaborators, 2022). Due to increased healthcare throughout the world, the proportion of older people in the population increases with time which in turn increases the total number of individuals with AD which is projected to rise from the current about 50 million to approximately 139 million by 2050. The major hallmark features of AD include the accumulation of extracellular amyloid beta (Aβ) plaques and the presence of intraneuronal neurofibrillary tangles of hyperphosphorylated tau in the brain (Hampel et al., 2021; Surguchov et al., 2023). These pathological features lead to neuroinflammation, proteostasis failure, synaptic dysfunction, and consequently loss of cognition and changes in personality (Mangalmurti and Lukens, 2022; Peng et al., 2022; Morrone et al., 2023).

Alzheimer’s disease is a complex, highly heterogeneous and heritable trait (Gatz et al., 2006). Although the genetic cause of familial AD (FAD) has been identified and well characterized through highly penetrant variants in *APP* (Goate et al., 1991), *PSEN1* (Sherrington et al., 1995), and *PSEN2*, (Levy-Lahad et al., 1995), these autosomal dominant forms account only for less than 1% of AD cases (Bekris et al., 2010). Thus the cause of more than 99% of cases of late-onset AD (LOAD) still needs to be identified and studied. Interestingly, rare coding variants in *PSEN1* and *PSEN2* have also been found in many families with LOAD (Cruchaga et al., 2012) suggesting a genetic continuum between FAD and LOAD. The first family based studies that identified the apolipoprotein E (*APOE*) ε2 and ε4 alleles with two missense mutations is the strongest risk factor across genome-wide association studies (GWAS) of AD (Jansen et al., 2019; Kunkle et al., 2019). The success of the *APOE* case-control association design led to more studies such as the rare variant association studies (Kamboh, 2018), small samples of whole exome sequence (WES) studies (Guerreiro et al., 2013), Large GWAS of common variants (Kunkle et al., 2019; Andrews et al., 2020), as well as the large-scale sequencing efforts like the Alzheimer’s Disease Sequencing Project (ADSP) (Beecham et al., 2017) which have all implicated dozens of loci but do not implicate the FAD genes.

Following the identification of numerous risk genes, the enrichment pathway analysis has identified 45 significantly enriched biological processes including the immune system (Lambert et al., 2010), APP and tau-related protein metabolism (Bellenguez et al., 2022), cholesterol efflux, negative regulation of autophagy, membrane organization, vesicle docking, endocytosis, and phosphorus metabolism among others (Xue et al., 2021). The autophagy–lysosomal pathway (ALP) is involved in the degradation of long-lived proteins, and reduced ALP activity during aging results in protein aggregation and the generation of toxic protein species (Davoody et al., 2023; Ou-Yang et al., 2023). Based on known genetic risk factors, Aducanumab is the first FDA-approved amyloid-lowering immunotherapy developed for AD followed by Lecanemab. However, recent post-marketing data show that amyloid-related imaging abnormalities (ARIA) such as ARIA-E (edema) or ARIA-H (hemorrhage) occur in about 25% of participants, all APOE-ε4 carriers, treated with these antibodies. Treatment was discontinued in 4 out of 24 cases of moderate-severe ARIA-E (Agarwal et al., 2023; Howe et al., 2023). Thus, it is crucial to identify more AD risk factors and characterize them to unravel novel biological pathways for future targeting.

Here we focused on leucine-rich repeat-containing protein 25 (LRRC25) since it was recently shown to be within the AD risk loci by sequencing the transcriptome of microglia and analyzing chromatin accessibility profiling in primary human microglia from 150 AD donors (Kosoy et al., 2022). LRRC25 is a potential single-pass type I membrane protein and has 4 leucine-rich repeats, a glycosylation site, and an F-box domain that interacts with the E3 ubiquitin ligase, participating in ubiquitin-proteasome system (UPS) for protein degradation (Ng et al., 2011). LRRC25 is known to be mainly expressed in immune cells such as monocytes, granulocytes, dendritic cells, and T lymphocytes. Functionally, LRRC25 is implicated in regulating autophagy during viral infection and has been shown to promote the degradation of RIG-1 and p65/RelA (Du et al., 2018) thereby negatively regulating the signaling pathways of NF-κB (Feng et al., 2017), and interferon (Du et al., 2018) and thus suppress the production of inflammatory cytokines. Additionally, LRRC25 was confirmed to play a protective role in primary lower-grade glioma (Zhang et al., 2020), and was shown to be significantly upregulated during neonatal hypoxic-ischemia neuronal damage *in vitro* (Xiong et al., 2020). However, whether LRRC25 is expressed in neurons of the brain and contributes in any way to AD has not been explored. Here using cell-type specific antibodies we show that LRRC25 is expressed in neurons, astrocytes, and microglia in the mouse brain as well as cell lines and most importantly show a robust increase in LRRC25 protein levels in the mouse models of AD, AD patient brains, and iPSC-derived neurons from AD patients.

## Materials and methods

### Chemicals and antibodies

The protease inhibitor microcystin-LR (cat# 475815) was purchased from Calbiochem-Millipore (Temecula, CA, USA). The dithiothreitol (cat # D9779), sodium orthovanadate (cat # 450243), and protease inhibitor cocktail (cat # P8340) to prepare lysis buffer were purchased from Sigma Aldrich (St. Louis, MO, USA). Nonidet-P40 substitute (cat # M158) to prepare NP40 lysis buffer was obtained from Amresco (Solon, OH, USA). USDA-certified fetal bovine serum (FBS) for cell cultures was purchased from BioFluid Technologies (cat # SKU: 100-500-Q). The PageRuler™ Prestained Protein Ladder, 10–180 kDa (cat # 26617), SuperSignal™ West Pico PLUS Chemiluminescent Substrate (cat # 34578), and B27 supplement for neuronal growth (cat # A365111) were purchased from Thermo Fisher Scientific. NuPAGE™ LDS Sample Buffer (4X) was purchased from Fisher Scientific (cat # NP0007). Monoclonal LRRC25 antibody (cat # sc-514216) and monoclonal actin antibody (cat # sc-47778) were purchased from Santacruz. Polyclonal LRRC25 antibody, (cat # PA5-106995) was purchased from Thermo Fisher, The cell-type specific antibodies such as GFAP Monoclonal Antibody (2.2B10) was purchased from Thermo Fisher Scientific (cat # 03-0300). Anti-NeuN Antibody, clone A60 (cat # MAB377). Mouse monoclonal anti-MAP2 antibody (cat # M9942-200UL) was purchased from Millipore Sigma (St. Louis, MO, United States). Anti-IBA1 polyclonal rabbit antibody (cat # 019-19741) was purchased from FUJIFILM, Wako Pure Chemical, Japan. Secondary antibodies such as peroxidase-conjugated AffiniPure goat anti-rabbit (code # 111-035-144) IgG (H+L) and goat anti-mouse (Code # 115-035-146) were purchased from Jackson ImmunoResearch Laboratories (West Grove, PA, USA). The Donkey F(ab’)2 anti-mouse IgG H&L (Alexa Fluor^®^ 568) (cat # ab175699) and donkey anti-Mouse F(ab’)2 IgG–H&L (DyLight^®^ 650), pre-adsorbed (cat # ab98769) for immunocytochemical staining were purchased from Abcam. The donkey anti-Rat IgG (H+L) Highly Cross-Adsorbed Secondary Antibody, Alexa Fluor™ 488 (cat # A-21208), donkey anti-Rabbit IgG (H+L) Highly Cross-Adsorbed Secondary Antibody, Alexa Fluor™ 488 (cat # A-21206), donkey anti-Mouse IgG (H+L) Highly Cross-Adsorbed Secondary Antibody, Alexa Fluor™ 488 (cat # A-21202) and donkey anti-Rabbit IgG (H+L) Cross-Adsorbed Secondary Antibody, DyLight™ 650 (cat # SA5-10041) were all purchased from Thermo Scientific. DAPI Fluormount-G (cat # 0100-20) for mounting slides was purchased from Southern Biotech (Birmingham, AL, USA). For immunoblot analysis, a 5% Americanbio Inc non-fat dry milk (cat # NC0115668), Fisher Scientific (Waltham, MA, USA) prepared in tris-buffered saline with 0.1% Tween-20 (TBS-T) was used to dilute all the primary antibodies, while the secondary antibodies were diluted directly in the 1X TBS-T buffer.

### Quantification of proteins by western blotting

All animal regulations were strictly enforced as per the latest edition of the National Institute of Health’s “Guide for the Care and Use of Animals and approved protocols by the Animal Care and Use Committee (IACUC) at Florida International University (FIU).” Two mouse models of AD, i.e., 3xTg (cat # 034830) and APΔE9 mice (cat # 034829) were purchased from Jackson Laboratories. The 3xTg mice are homozygous for all three mutant alleles homozygous for the Psen1 mutation and homozygous for the co-injected APPSwe and tauP301L transgenes [Tg(APPSwe,tauP301L)1Lfa, MMRRC stock #34830] (Oddo et al., 2003). APΔE9 mice (MMRRC Strain #034833-JAX) overexpress chimeric mouse/human APP (Mo/HuAPP695swe) and a mutant human presenilin 1 (PS1-ΔE9), both transgenes driven by independent prion promoters (Jankowsky et al., 2001). The genotype of the APΔE9 mice was confirmed initially by genotyping the tail genomic DNA and PCR analysis using specific primers. We used C57BL/6 as wild-type (WT) control mice. AD and NC brain tissues (hippocampus in all cases) were obtained from the “Harvard Brain Tissue Resource Center”, which is supported in part by PHS grant number R24MH068855. To quantify changes in LRRC25 protein levels during aging in the mouse brain, we used both male and female mice of 1 day (1D), 1 month (1M), 1 year (1Y), 1.5 years (1.5Y), and 2 years old (2Y) mice, all in C57BL/6 background. After euthanasia by carbon dioxide overdose, mice were decapitated, and cortex (CX), hippocampus (HP), brainstem (BS), and cerebellum (CB) were rapidly dissected and separated on ice and placed into lysis buffer (1% NP40 buffer with complete protease inhibitor mix) supplemented with sodium vanadate and microcystin. After homogenization, the brain lysates were subjected to centrifugation at 14,000 rpm for 20 min at 4°C. The lysate samples were mixed with equal amounts of NuPAGE™ LDS sample loading buffer, loaded into each well, and subjected to SDS-PAGE electrophoresis exactly as described previously (Lakshmana et al., 2009; Wang et al., 2013, 2022). The proteins were then transferred onto PVDF membranes, blocked with 5% milk prepared in 1% TBS-T buffer, and incubated overnight with primary antibodies at 500–1000 dilution followed by 1- to 2-h incubation with HRP-conjugated anti-rabbit or anti-mouse secondary antibodies in 1X TBS-T buffer. The protein signals were detected at different exposure times following incubation with the super signal west pico chemiluminescent substrate. Quantification of Western blot signals was done using ImageJ software. Actin signals were used to normalize protein levels in each sample and the protein levels were shown in percent of 1D control for aging studies and wild-type (WT) controls for AD mouse models.

### iPSC-derived neuronal cultures and differentiation into neurons

We purchased human iPSC-neural stem cells (NSCs) derived from dermal fibroblasts of AD patients with presenilin mutation (PSEN1 A246E) (cat # ax0114) and normal control (NC) human iPSC-neural stem cells (cat # ax0018) from Axol Bioscience Inc., and cultured them following manufacturer recommended protocol with slight modifications. Briefly, cell culture dishes were coated with Surebond (cat # ax0041) at 37°C for 4 h to promote attachment and growth of neural stem cells. The cryopreserved NSCs were thawed rapidly, mixed with neuronal medium, plated, and cultured in a humidified incubator with 5% CO2 at 37°C in the presence of epidermal growth factor at 20 ng/mL concentration and basic fibroblast growth factor at 20 ng/mL concentration. After few days, the cells were incubated with neural differentiation medium with supplements such as 1% Glutamax, 2% B27 and differentiation supplement. The medium was changed three times per week and maintained up to 4 weeks in culture. The neuronal phenotype was confirmed by immunocytochemical staining using NeuN and MAP2 antibodies as described above.

### Immunocytochemical staining and immunohistochemistry

For immunocytochemical localization of LRRC25, we first obtained cell lines such as astrocytoma (cat# CCF-STTG1), HMC3 (cat # CRL-3304), and Ntera-2 cells (NT2) cells (cat # CRL-1973), all from ATCC (Manassas, VA, USA) as cellular models of astrocytes, microglia and neurons, respectively. Briefly, on the second day of plating cells on coverslips, cells were washed three times with 1X PBS, fixed with paraformaldehyde (PFA) for 10 min, followed by three washes with 1X PBS and permeabilization in tris-buffered saline with 0.1% Tween 20 detergent (TBST) and then blocked with a blocking solution (normal donkey serum, 1%; BSA, 3%; gelatin, 1%; Triton X-100, 0.2%; saponin, 0.2%) for 30 min. Immunostainings were performed by incubating cells with LRRC25 antibody at 1:100 dilutions overnight. This was followed by incubation with Alexa Fluor 568-conjugated anti-rabbit IgG secondary antibody for 1 h followed by mounting with 4′,6-diamidino-2-phenylindole (DAPI) containing Fluormount-G (SouthernBiotech, Birmingham, AL, USA) to visualize the nuclei. Cells positive for LRRC25 were visualized under the cy3 (red) channel and images were captured in a BZX700 All-in-One microscopy system (Keyence Corp, Itaska, IL, USA). For immunocytochemical staining of iPSC neurons, we grew neurons for up to 16 days *in vitro* (16DIV) and followed the same steps as described above for cell lines. For double staining, we used LRRC25 rabbit polyclonal antibody and Neun mouse monoclonal antibodies. After the primary antibody was incubated overnight, secondary antibodies such as Donkey anti-Rabbit IgG (H+L) Highly Cross-Adsorbed Alexa Fluor™ 488 and the Donkey F(ab’)2 anti-mouse IgG H&L (Alexa Fluor^®^ 568) antibodies were incubated for 1 h followed by mounting with 4’,6-diamidino-2-phenylindole (DAPI) containing Fluormount-G (SouthernBiotech, Birmingham, AL, USA) to visualize the nuclei.

For immunohistochemical staining of mouse brain tissues, after euthanasia with isoflurane, the mice were fixed intracardially in 4% PFA prepared in 1X phosphate-buffered saline (PBS) using a perfusion pump, the brains were removed and cryoprotected in 30% sucrose in PBS for 3 days or until the brains were completely sunk. Then the whole brains were frozen in a Tissue-Tek OCT compound on a slab of dry ice. A 15-μm coronal brain section was cut in a cryostat (Leica) at −19 to 21°C. Tissue permeabilization was carried out using 0.4% Triton X-100 prepared in 1XPBS for 10 min. Non-specific binding was blocked by incubation in the blocking solution prepared as described above for 30 min. Primary antibodies such as anti-LRRC25, anti-GFAP, anti-IBA1, and anti-Neun prepared in a blocking solution (1:100 dilution) were incubated overnight at 4°C with gentle shaking. Secondary antibodies such as donkey anti-Rabbit IgG (H+L) Secondary Antibody DyLight™ 650, donkey anti-Rat IgG (H+L) Secondary Antibody Alexa Fluor™ 488, and donkey anti-Mouse IgG (H+L) Secondary Antibody, Alexa Fluor™ 488 were incubated 1 h at room temperature in the dark. The staining was followed by a final autofluorescence elimination step of incubation in an undiluted autofluorescence eliminator reagent (cat # 2160, EMD Millipore) for 1 min under vigorous shaking to prevent reagent precipitation. Finally, the slides were mounted with 4’,6-diamidino-2-phenylindole (DAPI) containing Fluormount-G (Southern Biotech, Birmingham, AL, USA) to visualize nuclei, and fluorescence signals were imaged and captured in a BZX700 All-in-One microscopy system (Keyence Corp., Itaska, IL, United States).

### Quantification of fluorescence intensity

We used ImageJ software to quantify LRRC25 fluorescence intensity in the NC- and AD-derived iPSC neurons. First, the microscope-acquired images were converted into RGB color, the scale was set in pixels, then by using the freehand tool the area of neurons was selected and then the fluorescence intensity was measured in numerical value. The fluorescence intensity was averaged from a total of 80–90 independent neurons for each of NC and AD.

### Statistical analysis

Statistical analyses were performed using the GraphPad Prism Software version 9.5.1 (GraphPad, San Diego, CA, USA). For comparisons in the levels of LRRC25 between two groups such as WT vs. 3xTg WT vs. APΔE9, or NC vs. AD human brains the student’s *t*-test with two tail parameter was used. Since the aging study involved different age groups in multiple brain regions, a one-way analysis of variance (ANOVA) followed by Tukey’s multiple comparison test was used. Data presented are the mean + standard error of the mean (SEM) and were considered significant only if *p* < 0.05. * indicates *p* < 0.05, **indicates *p* < 0.01 and ***indicates *p* < 0.001.

## Results

### LRRC25 is expressed in brain-relevant cell lines

**FIGURE 1:**
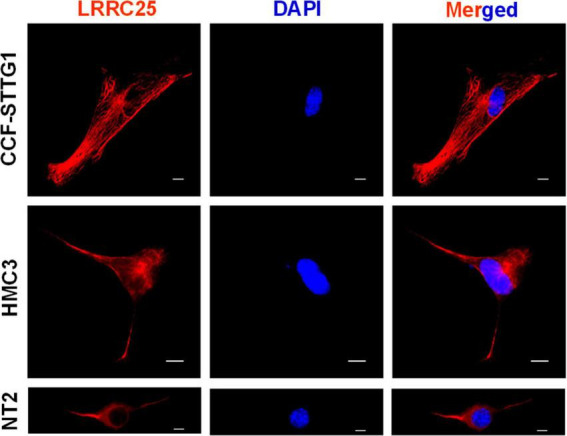
LRRC25 is expressed in astrocyte-like cells, microglia, and neuronal cell lines. The CCF-STTG1, HMC3, and undifferentiated NT2 cells were immunocytochemically stained with LRRC25 antibody and visualized the subcellular LRRC25 expression. There is a clear indication of the membranous localization of LRRC25 and punctuate appearance in the cytoplasm (red) in all three cell types, and DAPI-stained nuclei (blue) appear to show no LRRC25 signals. The scale bar is 10 μm.

We used human-derived astrocytoma, HMC3, and NT2 cell lines as cellular models of astrocytes, microglia, and neurons, respectively. CCF-STTG1 cells are astrocyte-like cells isolated from a patient with astrocytoma which have been successfully used by multiple investigators as a model of astrocyte (Elfakhri et al., 2019; Sawmiller et al., 2019; Tian et al., 2022). HMC3 is a microglial cell with a macrophage-like morphology isolated from the brain of a patient and has been authenticated by multiple investigators (Madsen et al., 2023; Pang et al., 2023; Tian et al., 2023). NT2 cells are clonally derived, pluripotent human embryonal carcinoma cell lines isolated from a male carcinoma patient (Lee and Andrews, 1986). NT2 cells express nestin, vimentin, and microtubule-associated proteins characteristic of neurons (Pleasure and Lee, 1993) and therefore are being widely used as an *in vitro* model of neurons. However, here we directly used NT2 cells with no differentiation by retinoic acid. The Morphology of each cell confirms its cell type as shown in Figure 1. From the ICC images, it is also clear that all three cell types show clear LRRC25 labeling in the plasma membranes but some punctuate staining is also seen in the cytoplasm of all three cell types (Figure 1). This suggests that LRRC25 may play a crucial role in all three cell types in the brain.

### LRRC25 is expressed in astrocytes, microglia, and neurons in the mouse brain

**FIGURE 2:**
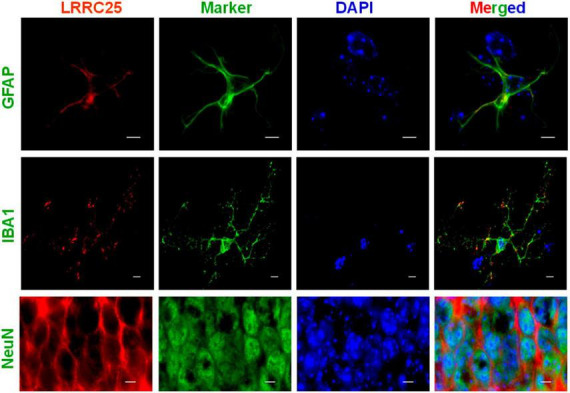
Demonstration of LRRC25 expression by astrocytes, microglia, and neurons in the adult mouse brains using cell-type specific markers. Polyclonal LRRC25 antibody was co-stained with monoclonal antibodies against GFAP (astrocyte), IBA1 (microglia), and Neun (neuron), followed by DAPI staining for nuclei (blue). There is a clear expression of LRRC25 (red) in the plasma membranes and a punctuate appearance in the cytoplasm of all three cell types examined, with a clear absence in the nuclei. The astrocyte and microglial cell images were acquired in the cortex region, whereas neuron images were acquired in the CA2 region of the hippocampus. The scale bar is 5 μm.

To confirm whether LRRC25 is also expressed in any specific type of brain cells *in vivo*, we used GFAP, IBA1, and NeuN antibodies to specifically label astrocytes, microglia, and neurons, respectively. First, the antibodies that we used labeled the specific cell types as confirmed by their expected morphology (Figure 2). Similar to results in cell lines in Figure 1, LRRC25 signals can be seen in the plasma membranes and also punctuate appearance in the cytoplasm of all three cell types studied. For the astrocytes (GFAP) and microglial cells (IBA1), we acquired images in the “cortex” region, whereas for the neurons (NeuN), we acquired images in the “CA2” region of the hippocampus (Figure 2). Thus, similar to cell lines, LRRC25 is expressed in all three cell types in the mouse brain.

### LRRC25 expression is both age- and brain region-dependent in the mouse brain

**FIGURE 3:**
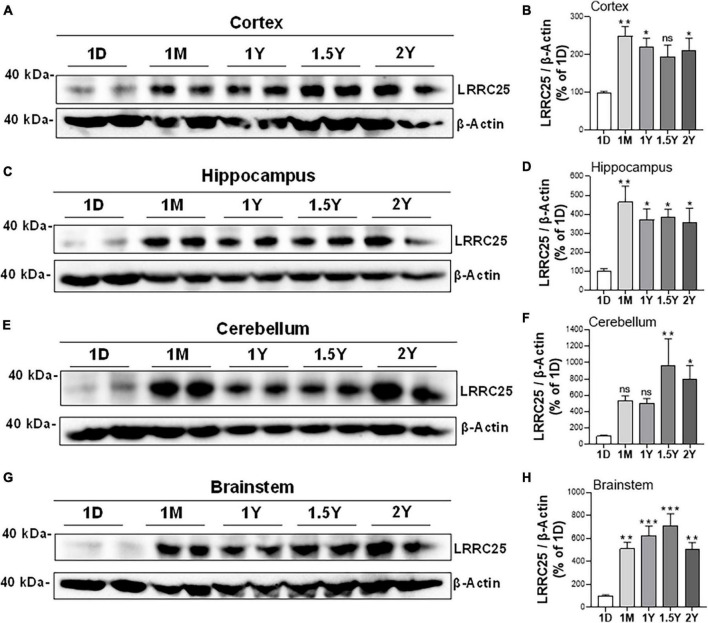
Differential expression levels of LRRC25 protein at different ages and in different brain regions. The brain regions such as cortex [CX, **(A,B)**], hippocampus [HP, **(C,D)**], cerebellum [BS, **(E,F)**], and brainstem [CB, **(G,H)**] were isolated on 1 day (1D), 1 month (1M), 1 year (1Y), 1.5 years (1.5Y), and 2 years (2Y) and brain lysates were subjected to immunoblotting. 1D expressed the lowest levels of LRRC25 in all brain regions, and relative expression levels at other ages were compared to 1D. The two lanes for each time point represent that samples were run in duplicates. Data were statistically analyzed by Analysis of Variance (ANOVA) followed by Tukey’s Multiple Comparisons Test. *p < 0.05, **p < 0.01, and ***p < 0.001, Data are mean + SEM, *n* = 3 per group.

Since there is no information yet on whether LRRC25 is expressed in the brain, and whether any changes occur during aging or if there is differential expression in brain regions, we quantified LRRC25 protein levels by immunoblots in the cortex (CX), hippocampus (HP), brainstem (BS), and cerebellum (CB) at different ages such as 1 day (1D), 1 month (1M), 1 year (1Y), 1.5 years (1.5Y), and 2 years (2Y) in the C57BL/6 wild-type (WT) mice. The two lanes for each time point represent that samples were run in duplicates. Results revealed that LRRC25 protein expression is the least at 1D in all of the brain regions studied such as CX. HP, BS, and CB (Figures 3A, C, E, G). Since 1D was the least expressed and earliest time point when LRRC25 was first detected, we used LRRC25 protein levels at 1D for relative comparisons among other ages. Thus, in the CX, LRRC25 expression levels were about 149% (*p* < 0.01), 121% (*p* < 0.05), 94%, and 112% (*p* < 0.05) at 1M, 1Y, 1.5Y, and 2Y, respectively (Figure 3B). Similarly, in the HP relative to LRRC25 protein levels at 1D, the levels were 364% (*p* < 0.01), 271% (*p* < 0.05), 286% (*p* < 0.05), and 255% (*p* < 0.05) in the 1M, 1Y, 1.5Y, and 2Y ages, respectively (Figure 3D). In the CB, however, only 1.5Y (867%, *p* < 0.01) and 2Y (698%, *p* < 0.05) ages were statistically significant (Figure 3F). Finally, in the BS, the increase in LRRC25 levels was about 413% (*p* < 0.01), 527% (*p* < 0.001), 612% (*p* < 0.01), and 403% (*p* < 0.05) in the 1M, 1Y, 1.5Y, and 2Y ages, respectively (Figure 3H). Thus in the CX, LRRC25 expression remains increased by more than twofold at all other ages compared to 1D. In the HP there is an increase of more than threefold at all ages relative to 1D. In both the CB and BS, the increase is even more and remains more than fourfold at all ages studied relative to 1D expression levels. Thus, overall based on the results presented in Figure 3, it is clear that LRRC25 expression in the adult ages remains higher than that of the postnatal period and also that there is no significant decrease at older ages such as 1.5Y and 2Y.

### LRRC25 protein levels are robustly increased in the APΔE9 and 3xTg mouse models of AD

**FIGURE 4:**
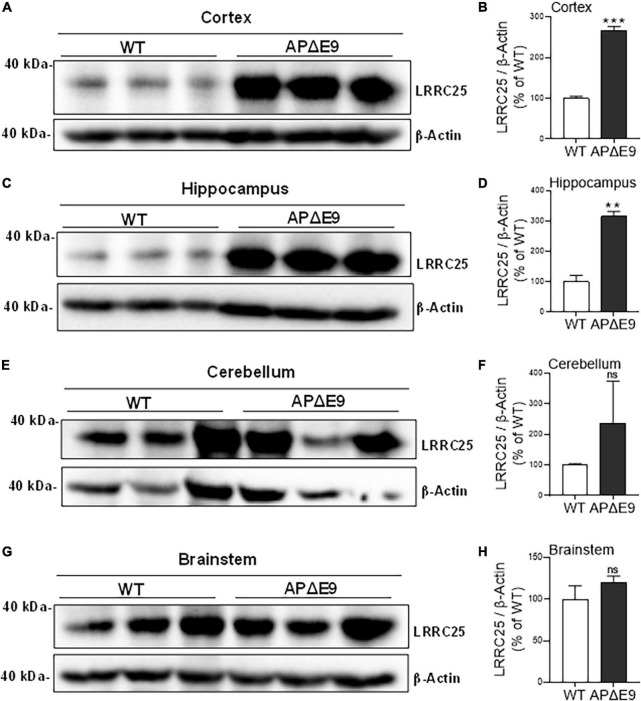
Different regions of APΔE9 mice show a robust increase in the levels of actin-normalized LRRC25 protein levels relative to wild-type (WT) controls. Relative to WT, the increased LRRC25 protein was 166.171% in the cortex [CX **(A,B)**] and 215.407% in the hippocampus [HP **(C,D)**], while the cerebellum [CB **(E,F)**] and brainstem [BS **(G,H)**] showed no significant (ns) changes in the APΔE9 mice relative to non-transgenic WT controls. Data were statistically analyzed by paired Student’s *t*-test. **p < 0.01 and ***p < 0.001, Data are mean + SEM, *n* = 3 per group.

**FIGURE 5:**
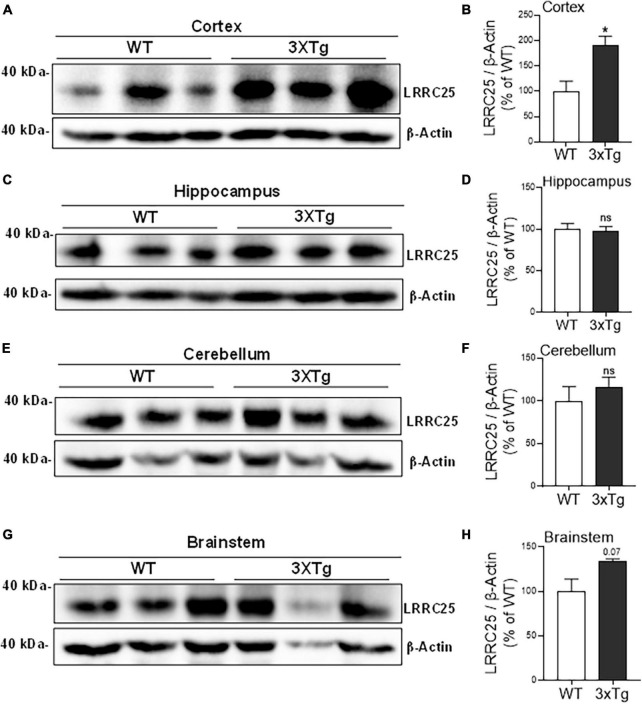
The cortical brain region of 3xTg mice shows significantly increased LRRC25 protein levels relative to wild-type (WT) controls. Image quantified and actin-normalized LRRC25 protein levels were compared among 3xTg and WT control mice. Relative to WT, the 3xTg mice cortex [CX **(A,B)**] showed 91.256% increased LRRC25 protein levels, but no changes in the hippocampus [HP **(C,D)**], cerebellum [CB **(E,F)**] and brainstem [BS **(G,H)**] were noted. Data were statistically analyzed by paired *t*-test. *p < 0.05. Data are mean + SEM, *n* = 3 per group.

Since a recent study suggested that LRRC25 may be a potential risk factor for AD, and since there is not much information about LRRC25’s role in the brain, as a first step, we wanted to quantify LRRC25 protein levels in the APΔE9 and 3xTg mouse models of AD. The APΔE9 mice overexpress chimeric mouse/human APP (Mo/HuAPP695swe) and a mutant human presenilin 1 (PS1-ΔE9), both transgenes driven by independent prion promoters. Because this transgenic line starts depositing plaques as early as 6 months and starts secreting Aβ within 3–4 months (Reiserer et al., 2007), this mouse line is a good model of early onset AD. Results showed that LRRC25 protein levels were significantly and robustly increased in the CX by 270% (**p < 0.001) (Figures 4A, B) and HP by 305% (**p < 0.01) (Figures 4C, D), while in both CB and BS, the LRRC25 levels were not significantly altered although an increased trend was noted in the APΔE9 brains when compared to the WT brains (Figures 4E–H). Having confirmed increased LRRC25 protein expression in the APΔE9 model of AD, we next wanted to test whether the increase in LRRC25 is specific to one model of AD or common to other models. Therefore, we also measured LRRC25 protein levels in the 3xTg mice. Unlike APΔE9 mice where only APP and presenilin mutations are driven to express, 3xTg mice also express tau P301L transgene, thus these mice are valuable for studying the impact of both amyloid and tau pathology (Oddo et al., 2003). Results revealed that similar to APΔE9 mice, CX showed a significant increase of LRRC25 protein by 185% (*p < 0.05) in the 3xTg mice compared to WT controls (Figures 5A, B). There was no significant change in the HP region (Figures 5C, D). The CB (Figures 5E, F) and BS (Figures 5G, H) showed an increased trend but it was not significant. Thus, two mouse models of AD showed increased LRRC25 protein in the CX brain region while the APΔE9 model also showed increased LRRC25 protein in the HP and BS. The CB is the only brain region among the studied regions where LRRC25 protein was not significantly altered, although an increased trend was noted particularly in the APΔE9 model of AD. This may indicate a pathogenic role of LRRC25 in AD consistent with a recent suggestion of its potential risk for AD.

### LRRC25 protein levels are also increased in the AD patients’ brains

**FIGURE 6:**
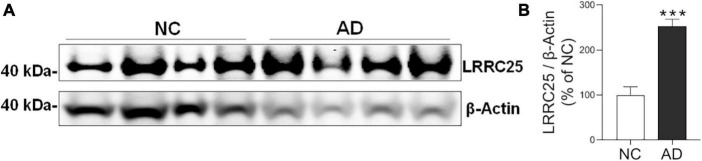
LRRC25 protein levels are robustly increased in Alzheimer’s disease (AD) brains. **(A)** Protein lysates were prepared from normal control (NC) subjects and AD patient brains, immunoblotted. **(B)** Quantification by ImageJ showed an increase of 153% in AD compared to NC. ***p < 0.001. Data are mean + SEM, *n* = 4 per group.

To confirm whether increased LRRC25 protein levels seen in mouse models of AD are reproducible in clinical settings, LRRC25 protein levels were also quantified in brain tissues from AD and normal controls (NC). The demographics of NC subjects and AD patients are given in Table 1. We used hippocampus brain regions from both NC and AD patient brains. Interestingly, and consistent with changes in AD mouse models, we found a significant increase of LRRC25 protein levels by 153% (*p* < 0.001) in the AD brains relative to NC brains (Figure 6). Thus, LRRC25 protein levels are consistently increased in both AD mouse models and AD patient brains.

**TABLE 1:** Demographics of the non-diseased controls (NC) and AD patients brain tissue donors.

	Gender (M/F)	Age (Y)	Avg Age (Y)	PMI (h)	Avg PMI (h)
NC-1	Female	58	77.5 ± 7.0	26.6	24.48 ± 3.49
NC-2	Female	89		14.12	
NC-3	Female	77		28	
NC-4	Female	86		29.18	
AD-1	Female	88	83.3 ± 1.7	17.07	14.43 ± 1.71
AD-2	Male	83		15.9	
AD-3	Male	80		9.42	
AD-4	Female	82		15.33	

The gender distribution of male/female (M/F), the average age in years (Y), and the postmortem interval (PMI) for tissue collection in hours (h). Data shown as mean ± SEM.

### iPSC-derived neurons from AD patients also show increased expression of LRRC25

**FIGURE 7:**
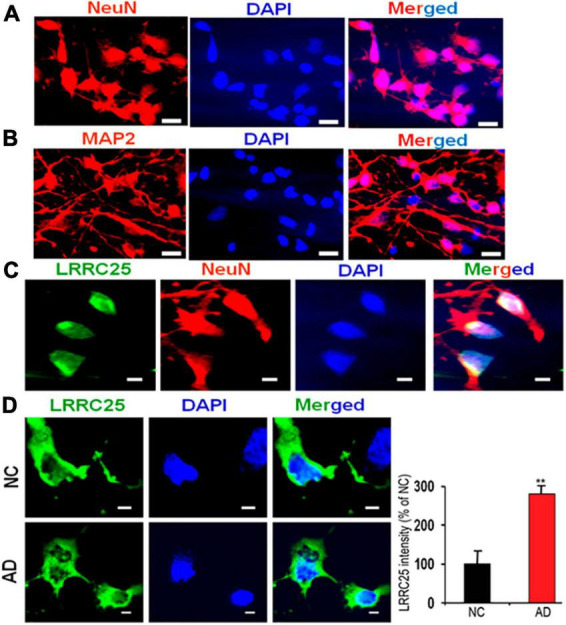
A robust increase in the LRRC25 fluorescence intensity in the fibroblast-derived iPSC neurons from AD patients when compared to iPSC neurons derived from normal control (NC) subjects. iPSC cells were grown and differentiated into mature neurons until 16 days *in vitro* (16DIV) and immunocytochemically stained for LRRC25, NeuN (red) as a marker of neurons, and MAP2 (red) as a marker of dendrites. **(A)** Shows Neun-positive mature neurons. **(B)** Shows MAP2-positive dendrites. **(C)** Positive staining of LRRC25 (green) in Neun-expressing cells (red) suggests LRRC25 is expressed in iPSC neurons. **(D)** Quantification of LRRC25 immunofluorescence showed an increase of 181% in AD-derived iPSC neurons when compared to NC-derived neurons. Data were statistically analyzed by paired *t*-test. **p < 0.01, Data are mean + SEM, *n* = 4 per group. The scale bar is 10 μm in **(A,B)** and 5 μm in **(C,D)**.

To understand whether LRRC25 is also altered in a more relevant cellular model of AD, we cultured and maintained iPSC-derived neurons from NC and AD patient fibroblasts as detailed in the section “Materials and methods.” After a complex network of neuritis was formed, we first confirmed the neuronal phenotype by immunostainings with NeuN, a marker of mature neurons, and MAP2 which stains dendrites at 16 days *in vitro* (16DIV) (Figures 7A, B). Further, by co-staining we confirmed LRRC25 protein expression in Neun-positive neurons. Importantly, to use iPSC neurons as a cellular model of AD, we also confirmed the expression of AD-related proteins such as APP and tau by staining with relevant antibodies (not shown). Even more importantly, quantification of LRRC25 immunofluorescence intensity showed a significantly increased level (181%, *p* < 0.01) in the AD-derived iPSC neurons when compared to NC-derived iPSC neurons (Figures 7C, D). These results are consistent with results shown in two AD mouse models (Figures 4, 5) and AD patient brains (Figure 6). The significance of the increased LRRC25 protein in AD models relative to NC needs to be further investigated.

## Discussion

Although LRRC25 has been recently shown to be a potential risk factor for AD through sequencing the transcriptome of microglia, how LRRC25 may contribute to AD pathogenesis is completely unknown. First of all, whether LRRC25 is expressed in neurons and brain is also unknown. Therefore, we initiated this study to analyze LRRC25 expression in multiple cell types of the brain in cell cultures, mouse and human brain parenchyma as well as iPSC-derived neurons. We found that LRRC25 is expressed in astrocytes, microglia, and NT2 cells, and most importantly we show that LRRC25 protein levels are increased in the APΔE9 and 3xTg mouse models of AD, AD patient brains, and iPSC-derived neurons from AD patient. Both APΔE9 and 3xTg mouse models of AD showed a robust increase in LRRC25 levels in the CX brain region. The APΔE9 mice also showed increased LRRC25 in the HP.

We are the first to demonstrate membranous and cytoplasmic expression of endogenous LRRC25 protein in the major brain cell types and therefore our results presented here cannot be compared with previous studies due to the lack of any such study. However, in a previous study, the cellular expression of LRRC25 was determined indirectly after ectopic transfection of GFP-LRRC25 in HeLa cells that were treated with TNF-α for 45 min and found punctuate appearance of LRRC25 in the cytoplasm (Feng et al., 2017). Since distinct cell types in the brain play different and specialized roles in the brain, LRRC25 expression in three major cell types suggests LRRC25 may be involved in multiple pathways and multiple functions.

Our finding that LRRC25 protein levels are increased in multiple models of AD cannot be compared with any other published data due to a lack of prior studies on the levels of LRRC25 in the NC and AD brains or cellular models. It should be noted that we used iPSC neurons derived from an AD patient with PSEN1 A246E mutation, while the brain tissues used were from AD patients confirmed by clinical pathology but whether they are cases of FAD or LOAD are unknown. However because LRRC25 levels were consistently increased in multiple models, LRRC25 may have a pertinent role in the etiology of AD. In this context, it should be noted that oxygen-glucose-deprived human fetal cortical neurons also showed increased LRRC25 mRNA and may suggest a crucial role in the pathogenesis of hypoxic-ischemic encephalopathy (Xiong et al., 2020). A prior study also reported that the expression of LRRC25 was significantly associated with the risk of developing breast cancer (Hoffman et al., 2017). Other pathological conditions that have been shown to upregulate LRRC25 protein but not mRNA levels include Foot-and-Mouth Disease Virus 3A Protein (Yang et al., 2020), vesicular stomatitis virus with enhanced GFP (VSV-eGFP), intracellular (IC) poly(I: C), and IFN-β (Du et al., 2018) and also LPS and TNF-α treatment (Feng et al., 2017). This evidence suggests that increased LRRC25 may have a pathological significance. On the contrary, LRRC25 levels are decreased in primary lower-grade glioma and many other tumor cell lines at both mRNA and protein levels (Zhang et al., 2020), and therefore may be protective against tumors. Currently, the mechanism by which LRRC25 levels were increased in AD is unknown. One possibility is that since autophagy is known to be impaired in AD with severe lysosomal acidification defects, the increased LRRC25 may be due to its reduced degradation. Future studies should determine whether increased LRRC25 occurs also at the mRNA level.

Another important role attributed to LRRC25 is in the autophagic degradation of RIG-1 by mediating the interaction between RIG-I and p62/SQSTM1 (the major autophagy receptor), thus LRRC25 may act as a secondary receptor in facilitating RIG-I delivery to autophagosomes for degradation in lysosomes in a p62-dependent manner (Du et al., 2018). LRRC25 has also been shown to promote p65/RelA degradation by autophagy (Feng et al., 2017). Crucial roles of many AD-associated genes in the autophagy-lysosome pathway (ALP), including presenilin 1, cystatin C, cathepsin D, and phospholipase D3 (Lee et al., 2010; Schuur et al., 2011; Hua et al., 2012; Cruchaga et al., 2014) suggest ALP pathway may play a central role in AD pathogenesis. Further, advancing age is the most prevalent risk factor for AD because of the decline of cellular protein quality control processes in the brain (Balch et al., 2008; Powers et al., 2009; Morawe et al., 2012) as evidenced by the accumulation of autophagosomes in AD brains (Cataldo et al., 1996; Nixon, 2007) which may be responsible for eliciting microglial activation and neuroinflammation. Whether LRRC25 plays any crucial role in autophagy in AD needs to be investigated. But given that multiple evidence suggest reduced ALP in AD brain as pointed out above, it is intriguing that increased LRRC25 protein levels observed in this study are expected to reduce protein accumulation based on the evidence that LRRC25 enhances the degradation of proteins such as RIG-1 and p65 by increasing autophagic degradation. If LRRC25 indeed enhances autophagic degradation, increased LRRC25 should reduce toxic protein accumulations in AD, i.e., amyloid plaques and neurofibrillary tangles. However, the hallmark feature of AD is the increased accumulation of these toxic proteins. It is also possible that increased LRRC25 levels may be in response to the accumulation of these toxic proteins, which may be insufficient to fully counter reduced ALP in AD. ALP is a complex process starting from the formation of phagophores to autophagosomes, followed by the fusion of autophagosomes with lysosomes to form autolysosomes, and finally degradation of cargo by the lysosomal enzymes. Each of these steps is regulated by multiple key proteins. It is also possible that if LRRC25 enhances the degradation of AD-related enzymes such as BACE1 or γ-secretase, then we expect complete abrogation of Aβ generation and therefore amyloid plaque formation. In this case, increased LRRC25 protein is expected to reduce AD neuropathology. On the contrary, if LRRC25 enhances the degradation of α-secretase like ADAM10, then it is expected to increase Aβ generation and therefore amyloid plaques. Future studies should investigate these possibilities.

In recent years neuroinflammation has emerged as a third hallmark feature of AD (Leng and Edison, 2021; Zhou et al., 2021; Mangalmurti and Lukens, 2022). LRRC25’s role in neuroinflammation in AD is also intriguing given that LRRC25 overexpression impairs and *LRRC25* knockout potentiates NF-κB activation thereby increasing the production of inflammatory cytokines (Feng et al., 2017), More recently LRRC25 has also been shown to inhibit IFN-γ secretion by microglia (Sheng et al., 2023). It is important to note that the anti-inflammatory role of LRRC25 has been demonstrated in response to viral infections. Therefore, the role of LRRC25 in inflammation may be context-dependent. In conclusion, here we provide preliminary evidence that LRRC25 protein levels are altered during aging in a brain region- and age-dependent manner and most importantly LRRC25 levels are increased in multiple models of AD. Whether and how LRRC25 may contribute to the pathogenesis of AD needs to be further investigated in future studies.

## Data availability statement

The raw data supporting the conclusions of this article will be made available by the authors, without undue reservation.

## Ethics statement

Ethical approval was not required for the studies on humans in accordance with the local legislation and institutional requirements because only commercially available established cell lines were used. The animal study was approved by Institutional Animal Care and Use Committee of Florida International University. The study was conducted in accordance with the local legislation and institutional requirements.

## Author contributions

DD: Data curation, Formal Analysis, Investigation, Methodology, Visualization, Writing – review and editing. JA: Data curation, Formal Analysis, Investigation, Methodology, Validation, Visualization, Writing – review and editing. MN: Conceptualization, Formal Analysis, Resources, Supervision, Writing – review and editing. ML: Conceptualization, Data curation, Formal Analysis, Funding acquisition, Methodology, Project administration, Resources, Supervision, Validation, Writing – original draft, Writing – review and editing.
